# Draft Genome Sequence of *Bacillus* sp. Strain NSP2.1, a Nonhalophilic Bacterium Isolated from the Salt Marsh of the Great Rann of Kutch, India

**DOI:** 10.1128/genomeA.00909-13

**Published:** 2013-10-24

**Authors:** Rinku Dey, Kamal Krishna Pal, Dharmesh Sherathia, Trupti Dalsania, Kinjal Savsani, Ilaxi Patel, Bhoomika Sukhadiya, Mona Mandaliya, Manesh Thomas, Sucheta Ghorai, Sejal Vanpariya, Rupal Rupapara, Priya Rawal, Anil Kumar Saxena

**Affiliations:** Microbiology Section, Directorate of Groundnut Research, Junagadh, Gujarat, Indiaa; Division of Microbiology, Indian Agricultural Research Institute, New Delhi, Indiab

## Abstract

The 5.52-Mbp draft genome sequence of *Bacillus* sp. strain NSP2.1, a nonhalophilic bacterium isolated from the salt marsh of the Great Rann of Kutch, India, is reported here. An analysis of the genome of this organism will facilitate the understanding of its survival in the salt marsh.

## GENOME ANNOUNCEMENT

The genomes of a number of species of *Bacillus* inhabiting the Little Rann and Great Rann of Kutch, India, have been sequenced recently with the aim of understanding their mechanism(s) of osmotolerance (1–3). *Bacillus* sp. strain NSP2.1 (16S rRNA GenBank accession no. JF802192), a nonhalophilic and endospore-forming bacterium isolated from the salt marsh of the Great Rann of Kutch, India, grows optimally without NaCl in the growth medium (NaCl range, 0 to 4.5%), at 37°C, and a pH 7.5. The genome of *Bacillus* sp. NSP2.1 was sequenced with the aim of understanding its survival mechanism(s) in the salt marsh.

The whole genome of NSP2.1 was sequenced by both shotgun and mate-paired library sequencing using the Roche 454 genome sequencer (GS FLX) at Macrogen Inc., South Korea, through Sequencher Tech Pvt. Ltd., Ahmedabad, India. In shotgun sequencing, 769,707 reads of 347,675,293 bases were obtained (average read length, 451 bp). The sequencing of mate-paired libraries generated 140,804 reads of 63,691,257 bp (average read length, 452 bp) and 140,783 reads of 64,402,285 bp (average read length, 457 bp).

We used the GS *de novo* assembler version 2.6 (4) for assembling the reads, with a coverage of 85-fold. The genome assembly of *Bacillus* sp. NSP2.1 (G+C content of 53.99%) contains 25 scaffolds and 107 contigs of 5,521,528 bp and 5,426,897 bp, respectively, with average lengths of 220,861 bp and 50,710 bp, respectively. An N_50_ scaffold length of 376,716 bp (smallest, 1,808 bp; largest, 1,854,053 bp), and an N_50_ contig length of 90,389 bp (smallest, 775 bp; largest, 380,501 bp) were obtained. All assembly data were deposited in the DDBJ/EMBL/GenBank nucleotide sequence database.

The draft genome of *Bacillus* sp. NSP2.1 was annotated by the RAST server (5), Glimmer 3 (6, 7), GeneMark (8, 9), the KEGG database (10), tRNAScan-SE (11), RNAmmer (12), and Signal P4.1 (13) for predicting subsystems, coding sequences (CDS), tRNA and rRNA genes, signal peptides, etc.

Using the different softwares, we predicted 5,425 coding sequences (CDSs), with 4,655,526 bp in the CDSs. There are 126 RNA-encoding genes (124 tRNA and 2 rRNA genes), 420 subsystems, and 329 signal peptides. Among the CDSs, 3,459 are not in the subsystem (1,405 nonhypothetical, 2,054 hypothetical), whereas 1,966 CDSs (1,871 nonhypothetical, 95 hypothetical) are in the subsystem. RAST annotation also revealed the involvement of 103 genes in stress responses: 10 in osmotic stress (1 in osmoregulation, 9 in choline and betaine uptake), 36 in oxidative stress (5 in protection from reactive oxygen species, 18 in oxidative stress, 2 in glutathione:nonredox reactions, 7 in redox-dependent regulation of nucleus processes, 1 in the glutathione:redox cycle, 2 in glutaredoxins, and 1 in glutathionylspermidine and trypanothione), 5 in cold shock, 15 in heat shock, 15 in detoxification, and 22 in no subcategory. A number of genes associated with ABC transporters (map02010), including osmoprotectants (OpuBC, OpuBB, and OpuBA), glycerol-3-phosphate uptake, and that of two-component systems (map02020), such as those involved in the response to K^+^-limitation and K^+^-transport, and salt stress-degrading enzymes, have been mapped.

Our laboratory is exploring the genome of *Bacillus* sp. NSP2.1 further to know the mechanisms of survival for this nonhalophilic bacterium in salty settings.

### Nucleotide sequence accession numbers.

This whole-genome shotgun project has been deposited at DDBJ/EMBL/GenBank under the accession no. AVBJ00000000. The version described in this paper is version AVBJ01000000.
